# Role of Malnutrition in Atrial Fibrillation: A Prospective Study including Individuals ≥ 75 Years of Age

**DOI:** 10.3390/nu15194195

**Published:** 2023-09-28

**Authors:** Kemal Göçer, Bayram Öztürk

**Affiliations:** 1 Department of Cardiology, Faculty of Medicine, Kahramanmaras Sutcu Imam University, Kahramanmaras 46050, Türkiye; 2 Department of Cardiology, Medical Park Goztepe Hospital, Istanbul 34730, Türkiye

**Keywords:** atrial fibrillation, malnutrition, elderly, GLIM criteria, CONUT

## Abstract

Background: Atrial fibrillation (AF) is the most common rhythm disorder in the elderly. The AF can cause life-threatening thromboembolic complications. Therefore, there is a need to determine the risk factors of AF. In this study, we aimed to examine the association of markers of malnutrition with AF in individuals aged 75 years and older and to find the factors that may affect mortality. Methods: In this prospective study, 358 consecutive individuals aged 75 years and older presenting to the cardiology outpatient clinic were included. All participants were divided into AF and sinus rhythm (SR) groups. In addition, a questionnaire and scoring system were used to assess malnutrition status. Information was obtained from all patients through outpatient clinic visits or telephone interviews for one year. Death from any cause was considered as the endpoint. Results: AF was observed in 71 (19.8%) patients. Death was higher in patients with AF (*p* < 0.001), high CONUT score (*p* = 0.018), and GLIM malnutrition (*p* = 0.018). GLIM malnutrition caused a 2.8-fold increase in the development of AF. Conclusions: Screening for malnutrition in the elderly is essential. According to GLIM criteria, malnutrition may play a role in the development of AF and increase one-year mortality in the elderly.

## 1. Introduction

The AF is the most frequent arrhythmia in elderly individuals [1]. In later life, pathophysiological processes affecting the atrium, such as dilatation and fibrosis of the atrium, lead to changes in the electrical state of the atrium, resulting in arrhythmia [2]. Once AF develops, the fibrotic process in the atrium accelerates further, forming a continual loop. AF is common in the elderly, with over 10% of patients aged ≥80 affected [3]. Apart from age, male gender, central obesity, thyroid condition, prior heart failure, or cardiovascular diseases are relevant to AF [4]. Patients with AF are five times more likely to have an ischemic stroke and twice as likely to have an all-cause mortality when compared to those without AF [5]. The reason for such a high risk of stroke is blood stasis due to loss of contractile function in the left atrium. Stasis of blood leads to thrombus formation and embolism complications [6]. The fact that such a life-threatening disease has become an epidemic with the aging of the population has increased health screening for AF and accelerated the development of medical treatments for AF [7]. AF can be diagnosed incidentally during routine examinations. However, especially in elderly and dependent patients, there are severe problems in treating and managing AF in underdeveloped countries where home care services are inadequate [8].

The average life expectancy of the elderly has increased due to technological developments in health, early recognition of diseases, and innovations in treatment modalities. The increasing elderly population has led to the need for a detailed examination of nutrition in the elderly [9]. Nutrition is crucial in protecting the elderly from diseases and prolonging quality of life and longevity. Inadequate nutrition has been shown to affect both physical and cognitive functioning in the elderly and increase the risk of chronic diseases [10,11]. Malnutrition can also be defined as poor nutrition in the elderly. It is caused by inadequate or excessive intake of nutrients. The physiology of aging is characterized by inadequate nutrient intake and increased inflammatory processes [12]. Therefore, malnutrition is an immense risk for the elderly. This is also called anorexia of aging. Aging anorexia includes changes in the gastrointestinal tract, cachexia, decreased sensory function, and sarcopenia [13]. There is also a decrease in bone mass, and osteoporosis is often seen [14]. The immune system cannot function properly. As a result, wound healing is delayed, and the protective shield against disease is removed. It predisposes to the development of sepsis [15]. All these processes that negatively affect health pose a significant problem for the elderly.

Elderly AF patients (≥75 years of age) have difficulty presenting to healthcare institutions due to multiple comorbid diseases and poor nutritional status. Consequently, they are not sufficiently represented in clinical trials [16]. Although geriatric evaluation is recommended in some guidelines for older AF patients, studies on malnutrition status and the relationship of malnutrition with AF prognosis are limited. Malnutrition is common in hospitalized patients. In hospitalized patients, malnutrition assessment is often overlooked. Assessing the nutritional status of patients with AF during hospitalization allows us to detect and treat malnutrition. Malnutrition treatment also contributes to improving the progression of patients by reducing the length of hospitalization and the financial burden [17]. Therefore, malnutrition may be treated in the elderly. By determining the malnutrition status of elderly patients with AF with nutritional assessment, early detection aims to prevent the adverse clinical consequences of malnutrition and improve their prognosis. Various non-objective systems and scores, such as mini-nutritional and subjective global assessments, have been proposed to evaluate malnutrition [18]. Nevertheless, PNI, CONUT, and GNRI are more objective scores and are often employed in clinical trials and practice [19,20]. Recent studies show a strong association between the scores of CONUT, PNI, or GNRI, which measure malnutrition, and the clinical outcomes of patients suffering from myocardial infarction, heart failure, and peripheral arterial disease [21,22]. In addition, the GLIM score, which emerged in 2019, is used for early diagnosis and treatment of malnutrition in some special populations, such as oncology. This score identifies patients at risk and assesses the severity of malnutrition [23]. Studies evaluating AF and malnutrition together are rare. In light of this information, we aimed to reveal the factors affecting the presence of AF in patients over 75 years of age and to determine the relationship between malnutrition and mortality in the elderly with AF.

## 2. Materials and Methods

### 2.1. Study Design

**Figure 1:**
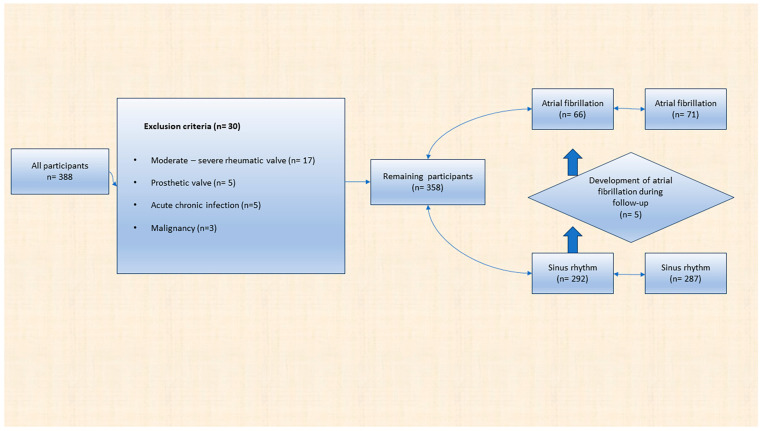
Flowchart of the study.

This prospective, cross-sectional study included 388 consecutive participants aged ≥75 years who presented to the cardiology outpatient clinic from three centers between June 2022 and August 2022. Thirty of these participants were excluded from the study. The study was conducted with the remaining 358 patients (Figure 1). Data were collected from all participants regarding developing adverse events for one year through outpatient clinic controls or phone calls. Patients were divided into atrial fibrillation (AF) and sinus rhythm (SR) groups. Demographic and clinical characteristics of all patients were recorded. Electrocardiography and echocardiography were performed. Blood was collected from all patients for laboratory parameters. All patients ‘ nutritional status, cognitive characteristics, and physical capabilities were assessed using scoring systems or questionnaires. All these values were compared between the two groups. In addition, patients were analyzed in two groups, the dead and survivors, after one year of follow-up. The primary endpoint was death from any cause. Factors affecting mortality were investigated. Finally, all patients were categorized according to high controlling nutritional status (CONUT) score and the presence of the global leadership initiative on malnutrition (GLIM). Nutritional status was compared between variables. Written informed consent was obtained from all patients for the study. The study was conducted in accordance with the Declaration of Helsinki and was approved by the Ethics Committee of Kahramanmaras Sutcu Imam University Faculty of Medicine, Turkey (protocol code 40 and date of approval 25 June 2022).

#### Exclusion Criteria

Thirty patients did not meet the criteria for the study. The exclusion criteria were as follows: moderate to severe rheumatic valve, prosthetic valve, acute or chronic infections, sepsis, malignancy, and life expectancy of less than three months.

### 2.2. Atrial Fibrillation Definition

Atrial fibrillation is the most common rhythm disorder in the elderly, characterized by irregular, chaotic beats in the atria, with an atrial rate ranging between 350–600 beats/min. On electrocardiography, the absence of evaluable *p* waves and irregular R-R intervals are diagnostic for AF [24]. The AF is classified into four groups: paroxysmal, persistent, long-standing persistent, and permanent AF. AF that spontaneously terminates (returns to SR) up to day seven after onset or is terminated (returned to SR) by electrical or pharmacologic cardioversion up to day seven after onset is paroxysmal AF. AF lasting seven days or more or terminated by cardioversion on or after day 7 is persistent AF. AF lasting more than 12 months, but in which it is decided to adopt a rhythm control strategy, is long-term persistent AF. AF in which no attempt to return to SR is considered and a rate control strategy is adopted is defined as permanent AF [24]. In patients with SR on electrocardiogram, irregular atrial beats for more than 30 s on 24-h Holter monitoring were considered AF.

### 2.3. Echocardiography

Echocardiography was performed in all patients according to European and American echocardiography guidelines [25,26]. Echocardiographic measurements of cardiac dimensions were obtained in the lateral or decubitus position. Color Doppler ultrasound was used for heart valves. PW Doppler evaluated diastolic mitral flow patterns. Pulmonary artery pressure (PAP) was measured via CW Doppler. Left ventricular ejection fraction (LVEF) was evaluated using Simpson’s method. The brand of echocardiographic equipment varied between the institutions included in the study, but two operators reviewed echocardiographic measurements.

### 2.4. Controlling Nutritional Status

CONUT is a nutritional index derived from albumin, total cholesterol, and lymphocyte count parameters. The serum albumin level indicates the protein reserve in the body. Total cholesterol represents the calorie expenditure of individuals. The total lymphocyte count is an indicator of the immune system. Malnutrition can lead to a decrease in lymphocytes due to a compromised immune system. Utilizing these three parameters to create a scoring system yields a total CONUT score. A high CONUT score indicates the severity of malnutrition [27]. CONUT parameters and scoring are presented in Table 1.

### 2.5. The Prognostic Nutritional Index

The prognostic nutritional index (PNI) assesses a person’s nutritional and immunological status. Research has evidenced that PNI can be a prognostic marker for malignancy, infectious diseases, and cardiovascular diseases. When the PNI score is lower, the risk of malnutrition is higher. The PNI score is calculated using the formula ‘10 × serum albumin (g/dL) + 0.005 × total lymphocyte count (mm3)’. Severe malnutrition was classified as PNI below 35, moderate malnutrition as PNI between 35–38, and PNI above 38 as normal [28].

### 2.6. The Geriatric Nutrition Risk Index

The Geriatric Nutrition Risk Index (GNRI) is a simple screening tool to estimate the risk of nutrition-related morbidity and mortality in older patients. This index was first reported by Bouillanne et al. Patients were divided into four groups according to GNRI: major risk group (GNRI: <82), intermediate risk group (GNRI: 82–<92), low-risk group (GNRI: 92–98), and no-risk group (GNRI: >98). The GNRI is also used to assess the prognosis of chronic diseases [29].

### 2.7. Barthel Index

This index evaluates the reliance of elderly patients on external aid to accomplish their daily physical activities and requirements. This index has ten questions, and the responses are based on the patient’s or their family’s opinions. It is divided into five categories: fully independent (≥100), partially dependent (91–99), moderately dependent (62–90), highly dependent (21–61), and fully dependent (0–20) [30,31].

### 2.8. FRAIL Scale

Frailty was assessed with the FRAIL scale. It consists of a questionnaire with five questions (fatigue, resistance, ambulation, illnesses, and loss of weight) named after its initials. The questionnaire with yes and no answers is practical and easy to assess. Frailty score was categorized as 0 normal, 1–2 pre-fragile, and 3–4 fragile [32].

### 2.9. Pfeiffer Questionnaire

This survey evaluates the cognitive abilities of older people and the effects of organic brain damage on them. The questionnaire covers verbal fluency, spatial cognition, arithmetic, and logical thinking. Parents or caregivers can provide information about the test. The scale consists of 10 questions. A high score indicates brain damage (maximum score: 10). Scoring is based on race and educational background. The scoring is as follows: 0–2, normal; 3–4, mild mental disorder; 5–7, moderate mental disorder; ≥8, severe mental disorder [33].

### 2.10. Sarcopenia

Sarcopenia is a significant component of physical frailty. It is assessed with the SARC-F screening tool, which can be easily identified in daily practice. The SARC-F is a questionnaire consisting of five questions: Strength (S), Assisted Walking (A), Rising from a Chair (R), Climbing Stairs (C), and Falls (F). Each question can be scored from 0 to 2. The recommended cut-off value is ≥4 points. A correlation between SARC-F and clinical outcomes has been established in elderly and chronic diseases [34].

### 2.11. Mini-Mental State Examination Score

Mini-mental state examination (MMSE) score can be utilized to ascertain cognitive impairment in acute or chronic illness. It assesses six mental abilities: orientation to time and place, attention/concentration, short-term memory, language skills, visual-spatial abilities, and the ability to understand and follow instructions. The MMSE was split into two parts: normal and anormal. A top score of 30 was attainable for the MMSE. A score of 25 or higher is classified as normal. If the score is below 24, the result is generally considered abnormal and indicates possible cognitive impairment [35].

### 2.12. Global Leadership Initiative on Malnutrition (GLIM) 

This consensus begins with a validated initial screening test to detect patients “at risk”. The Nutritional Risk Screening-2002 was used for the initial screening test. According to this test, a score of 3 or above indicated the individual was at risk of malnutrition. Individuals at risk of malnutrition were passed to the second stage. Assessing and grading the severity of malnutrition is the second step. This step is evaluated according to three physical criteria: low BMI, unintended weight loss, and a decrease in muscle mass, as well as two etiologic criteria—a decrease in food consumption or digestion and an inflammation/illness burden. According to GLIM criteria, at least one phenotypic criterion and one etiologic criterion must be present to diagnose malnutrition. Phenotypic criteria are used to grade the severity of malnutrition into stage 1 (moderate) and stage 2 (severe) [36].

### 2.13. Statistical Analyze 

All data were analyzed using SPSS 22.0 (SPSS Inc., Chicago, IL, USA) software. When individuals over 75 years of age were divided into AF and SR groups, the Mann–Whitney U test was used to compare continuous variables for those not conforming to normal distribution, and an independent samples *t*-test was applied for those conforming to a normal distribution. The normality of data distribution was assessed by calculating skewness and kurtosis statistics. Patients were also categorized into three groups according to the CONUT and GLIM nutritional index. Pearson correlation analysis was performed between malnutrition scores. In addition, patients were followed up for one year. Comparisons were also made between those who died and those who survived. Using Kaplan–Meier analysis, survival tables were created. Finally, a multivariate logistic regression analysis was performed for factors affecting the presence of AF. Bland–Altman plots were used to evaluate measurement bias. *p*-value < 0.05 was considered statistically significant.

## 3. Results

### 3.1. Evaluation of All Participants

**Figure 2:**
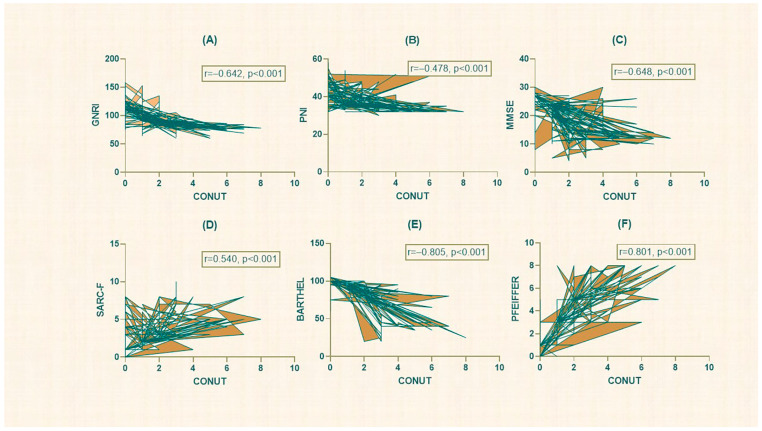
Correlation graphs for malnutrition and mental scoring systems. (**A**) CONUT-GNRI, (**B**) CONUT-PNI, (**C**) CONUT-MMSE, (**D**) CONUT-SARC-F, (**E**) CONUT-BARTHEL index, (**F**) CONUT-PFEIFFER Questionnaire.

A total of 358 participants were included in the study. Of these, 187 (52.2%) were male, and the mean age was 82.71 ± 5.83 years. Coronary artery disease was the most common disease in 55 (15.4%) participants. AF was observed in 71 (19.8%) patients. The mean CONUT score (1.54 ± 1.58), PNI (37.88 ± 5.19), GNRI (95.41 ± 16.49), Pfeiffer Questionnaire (3.37 ± 2.30), FRAIL scale (1.20 ± 1.01), Barthel index (84.83 ± 20.51), SARC-F (2.91 ± 1.88), MMSE (20.25 ± 6.08) were obtained in all participants. GLIM malnutrition was present in 139 (38.8%). Seventy-two (20.1%) patients had a high CONUT score. The correlation analysis between the participants’ scores is shown in Figure 2.

### 3.2. Atrial Fibrillation and Sinus Rhythm Group

Demographic and clinical characteristics of the AF and SR groups are shown in Table 2. Age (*p* = 0.583) was similar between the two groups, while male gender was higher in the AF group. Known diseases were similar between the two groups.

Biochemical and echocardiographic results of the groups are shown in Table 3. Among laboratory values, albumin (*p* = 0.010) and LVEF (*p* = 0.036) were lower in the AF group. LVEDD (*p* = 0.001) was higher in the AF group.

Table 4 shows the comparison of malnutrition and mental scores between the groups. Among the parameters assessed, only PNI (*p* = 0.122) was similar between groups.

### 3.3. The Presence of GLIM Malnutrition and High CONUT Score

Participants were divided into two groups, with and without malnutrition, according to GLIM criteria. They were also categorized based on high and low CONUT scores. AF was noticeably elevated in the high CONUT and GLIM malnutrition groups. Comparison with other parameters is expressed in Table 5.

### 3.4. 1-Year Survival Analysis

**Figure 3:**
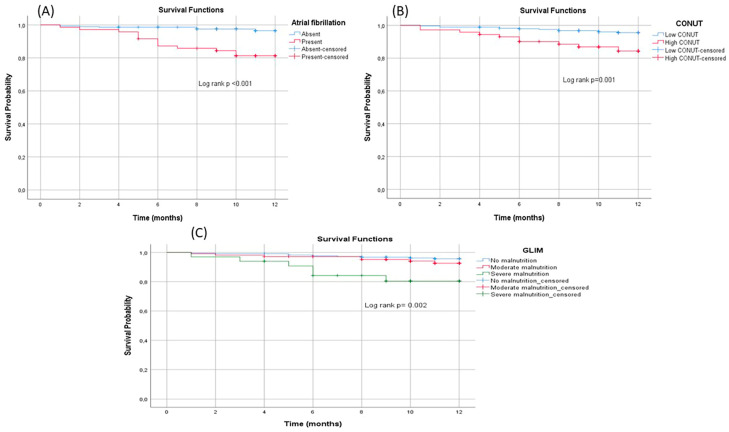
Kaplan–Meier survival curves of those with atrial fibrillation, GLIM malnutrition, and high CONUT score. (**A**) Survival analysis was performed in atrial fibrillation, (**B**) survival analysis of high CONUT, (**C**) survival curve of GLIM malnutrition.

During the 1-year follow-up period, 22 participants died from any cause. When the survivors and the dead groups were compared, the presence of AF (*p* ≤ 0.001) and low LVEF (*p* = 0.001) had the highest statistical significance in the dead group. The association with other parameters is shown in Table 6. In addition, the survival analysis of those with GLIM malnutrition, high CONUT, and AF is shown in Figure 3.

### 3.5. Factors Affecting Atrial Fibrillation

The presence of GLIM malnutrition, LVEF, albumin, hypertension, diabetes mellitus, chronic obstructive pulmonary disease, and GFR were included in the logistic regression analysis. The presence of GLIM malnutrition (B = 1.036, *p* < 0.001, OR = 2.819, 95% Cl = 1.613–4.927) and low LVEF (B = −0.107, *p* = 0.002, OR = 0.899, 95% Cl = 0.842) were found to be independent risk factors for AF (Table 7).

## 4. Discussion

The AF, characterized as chaotic atrial contraction, can cause severe complications in the elderly. Many risk factors for AF have been identified. Antiarrhythmic and anticoagulant drugs are used in the treatment of AF. Anticoagulant therapy could be the most critical aspect of treatment. The CHA_2_DS_2_-VASc score is the most commonly used scoring system for initiating anticoagulant therapy. According to this scoring, it is recommended that anticoagulant therapy be initiated in all patients with AF over 75 years of age to prevent cerebrovascular thromboembolism [37]. In particular, it is aimed to elucidate the risk factors of AF, which can cause fatal complications in patients over 75 years of age, and to improve the quality of life and longevity of the elderly. Malnutrition can be expected in the physiological processes of old age [1]. Cognitive and physical abilities can be reduced. Evidence has also been presented that it is linked to cardiac diseases [38]. In this study, we investigated the effect of nutritional status on the development of AF in the elderly. As a result of our study, an increase in the presence of GLIM malnutrition and LVEF was found to be an independent risk factor for the development of AF. There was a correlation between mental assessment and malnutrition indices. One-year mortality was higher in the presence of GLIM malnutrition, AF, and high CONUT score.

Adequate nutrition and diet are some of the most essential basic needs for disease prevention and healthy living in elderly patients. Loss of appetite causes malnutrition in elderly patients, leading to muscle loss and, consequently, weight loss [39]. A lack of the nutrients necessary to meet physiological needs can bring about heightened complications of chronic diseases, extended hospitalization, high infection rates, and even death [40,41]. Research has revealed that adequate nourishment plays a part in the recovery from disease and can lower the frequency of long-term illnesses. In addition, it has been stated that social support practices can identify the elderly at risk for malnutrition and solve their problems. Early intervention to prevent malnutrition can significantly reduce weight loss [42].

The literature has revealed that malnutrition is essential in the prognostic process. Generally, studies have utilized a single marker and method to define malnutrition. Therefore, diagnosing malnutrition using a single method may not produce accurate results [43]. The one-year mortality rate was 19.2% in a retrospective study with relatively few patients. However, in this study, the problems experienced by the elderly regarding health insurance were cited as the reason for such a high mortality rate. Frailty and sarcopenia were not assessed at hospital discharge in these patients. Therefore, the association of malnutrition with poor prognosis is not entirely clear [44].

The risk of malnutrition in patients with heart failure varies between 16% and 90% in studies. The broad spectrum of malnutrition rates in these reports is owing to distinctions in the medical characteristics of the patients and various definitions of malnutrition [45]. Sze et al. reported 8% malnutrition based on PNI and 54% malnutrition based on CONUT nutritional index in heart failure patients. Furthermore, standardization of the definition of malnutrition may lead to a failure to fully assess the impact of malnutrition on the elderly with chronic diseases [21]. There is a need for a globally accepted, standardized definition of malnutrition. Therefore, the GLIM criteria have recently been introduced. Malnutrition rates were 42% in patients with inflammatory bowel disease requiring surgical intervention. In addition, 25.8% of those with hematologic cancer and 24% with lung cancer were diagnosed according to GLIM criteria [46,47].

The extent of malnutrition may differ across different studies. Malnutrition was present in 80% of elderly patients with hypertension, and 65.7% of elderly patients with AF had malnutrition, as measured by at least one malnutrition score [48,49]. In a study, the prevalence of malnutrition calculated using CONUT, GNRI, and PNI scores in outpatients with heart failure was 57%. In an observational study, 28.3% of patients who underwent multiple percutaneous coronary interventions had malnutrition [21]. In our study, the malnutrition rate was 38.8% according to GLIM criteria. Applying a two-step system to identify malnutrition according to GLIM criteria might result in lower malnutrition rates.

Oral nutritional supplements positively affect energy intake and body weight in elderly patients with malnutrition. A sufficient diet for those who have undergone cardiac surgery has been found to correlate with an earlier discharge and more rapid recovery [50]. Studies have demonstrated that proper nutrition or adding nutritional supplements can improve the function of the left ventricle and the quality of life for those suffering from long-term heart failure [51]. Additional research is necessary to assess the impact of dietary aid or supplementation on clinical results in elderly AF sufferers.

Adverse clinical outcomes may occur in AF patients due to chronic disease, partly caused by malnutrition and arrhythmia. Malnutrition is a determinant of disease severity and causes the disease to become chronic. Chronic diseases also can result in malnutrition. Hypoalbuminemia is caused by AF, which triggers inflammation, leading to enhanced protein degradation and reduced protein synthesis. Blood contains albumin as its most plentiful protein, and it is responsible for controlling osmotic pressure and partaking in the transportation of drugs in the bloodstream. Studies have shown that low albumin levels increase cardiovascular disease and mortality rates [52]. Chronic inflammation suppresses normal immune and cellular functions due to decreased essential amino acids and vitamins. The number of cellular lymphocytes decreases, and as a result, the immune system is impaired. Lymphocytes are essential regulators of immunologic and inflammatory diseases in the body. Hypoalbuminemia and low lymphocyte count are associated with unfavorable clinical outcomes in elderly persistent AF patients. As a result of sarcopenia due to malnutrition, patients develop limitations in physical movements, leading to worse clinical outcomes. Patients, therefore, become frail. Frailty is associated with increased mortality and extended hospital stays, common in older AF patients. With aging, deterioration in organ functions, inadequate energy intake, and food access problems may occur. In elderly patients with oral malnutrition, the risk of systemic thromboembolism increases independently of other causes due to restriction in drug (especially anticoagulant) intake [53]. In our study, hypoalbuminemia was found in the AF group. However, it was not among the factors affecting AF.

The vicious circle between chronic diseases and malnutrition can explain the association of malnutrition with AF in elderly patients. AF is a chronic disease and is accompanied by an inflammatory process. Protein synthesis decreases and predisposes to hypoalbuminemia with the activation of inflammatory processes [54]. The chronic inflammation triggered by AF causes disruptions in the supply of essential nutrients (amino acids and vitamins) required for immune cells to function. As a consequence, malnutrition can occur. The opposite is also true. In the case of malnutrition, increased levels of many cytokines and interleukins involved in inflammation have been found [55]. This has led to the belief that malnutrition has similar pathological effects as chronic diseases. Malnutrition and chronic diseases are two intertwined conditions. A so-called cholesterol paradox has been reported in patients with AF [56]. Increased thromboembolic complications and mortality have been found in underweight AF patients. Our study suggests that malnutrition may accelerate inflammatory processes in the atria and cause chaotic contraction.

The high CONUT score in some patients is due to statin therapy’s low serum cholesterol levels. Therefore, the CONUT score overestimates malnutrition in patients receiving statin therapy. PNI is calculated by serum albumin level and lymphocyte count, reflecting immunologic nutritional status. In addition, the PNI score does not include mild malnutrition and, therefore, overestimates moderate and severe malnutrition. Since the PNI and GNRI scores do not include cholesterol compared to the CONUT score, they show less malnutrition in patients receiving statin therapy. Therefore, the GNRI score seems more appropriate to determine malnutrition in our study. Regardless of the malnutrition scores used in our study, malnutrition in elderly AF patients was associated with unfavorable clinical outcomes. However, in our study, GNRI and PNI were similar between the dead and survivor groups, whereas the CONUT score was higher in the dead group. This was because no participants were receiving statin treatment in the dead group.

Our study has some limitations. First, this study included elderly people in a specific region. Different results may be obtained in different races. Secondly, the patients’ socio-economic conditions were not considered in this study. Therefore, it may not be generalizable to the whole population. Third, the participants’ nutritional indices were assessed during inclusion. Changes within one year were not taken into account.

## 5. Conclusions

Malnutrition in the elderly may lead to an increased likelihood of AF. Calculation of malnutrition risk scores is essential in the clinical examination of patients. Improving malnutrition status is important in preventing the development of AF, which can cause serious problems, especially in the elderly. Our study showed that malnutrition predisposes to the development of AF. In addition, an increase in 1-year mortality was observed in those with AF and malnutrition defined according to GLIM criteria.

## Figures and Tables

**Table 1:** CONUT score formula.

Score	Serum Albumin (g/dL)	Total Lymphocyte Rate	Total Cholesterol (mg/dL)
0	≥3.5	1600	≥180
1		1200−1599	140−179
2	3.0−3.4	800−199	100−139
3		<800	<100
4	2.5–2.9		
6	<2.5		

Total score: ≤2 low, ≥3 high CONUT score.

**Table 2:** Demographic and clinical characteristics.

	Presence of AF N = 71	Sinus Rhythm N = 287	*p*-Value
Age, year	83.05 ± 5.89	82.63 ± 5.83	0.583
Male, *n* (%)	45 (63.4)	142 (49.5)	**0.036**
Current smoker, *n* (%)	9 (12.7)	20 (7.0)	0.115
BMI, kg/m²	24.36 ± 3.38	24.25 ± 3.21	0.806
GFR, mL/min/1.73 m^2^	67.49 ± 15.59	66.74 ± 16.75	0.733
Hypertension, *n* (%)	7 (9.9)	47 (16.4)	0.169
Diabetes mellitus, *n* (%)	3 (4.2)	25 (8.7)	0.208
CAD, *n* (%)	9 (12.7)	46 (16.0)	0.483
COPD, *n* (%)	11 (15.5)	33 (11.5)	0.359
Stroke, *n* (%)	5 (7.0)	16 (5.6)	0.638
Heart failure, *n* (%)	6 (8.5)	16 (5.6)	0.366
NYHA classification			
I	11 (15.5)	60 (20.9)	0.358
II	42 (59.2)	150 (52.3)	
III	13 (18.3)	66 (23.0)	
IV	5 (7.0)	11 (3.8)	
Usage drug			
ACE-inhibitors, *n* (%)	4 (5.6)	21 (7.3)	0.618
B-blockers, *n* (%)	18 (25.4)	77 (26.8)	0.801
Spironolactone, *n* (%)	1 (1.4)	11 (3.8)	0.310
Ca-channel blockers, *n* (%)	5 (7.0)	31 (10.8)	0.346
Furosemide, *n* (%)	5 (7.0)	23 (8.0)	0.785
ARB, *n* (%)	3 (4.2)	26 (9.1)	0.181
Statin, *n* (%)	8 (11.3)	40 (13.9)	0.554
Digoxin, *n* (%)	13 (18.3)	7 (2.4)	**<0.001**

Abbreviations: ACE, angiotensin-converting-enzyme; ARB, angiotensin receptor blocker; BMI, body mass index; CAD, coronary artery disease; COPD, chronic obstructive pulmonary disease; NYHA, New York Heart Association. Results in bold are significant (*p* < 0.05).

**Table 3:** Laboratory and echocardiographic parameters.

	Presence of AF N = 71	Sinus Rhythm N = 287	*p*-Value
Hemoglobin, g/dL	11.85 ± 1.37	11.74 ± 1.61	0.582
Neutrophil, 10^3^/µL	9.23 ± 3.59	9.62 ± 3.45	0.396
Lymphocyte, 10^3^/µL	2.55 ± 0.80	2.60 ± 0.90	0.633
Platelet, 10^3^/µL	280.18 ± 104.29	279.72 ± 99.07	0.972
Creatinine, mg/dL	1.07 ± 0.25	1.12 ± 0.26	0.221
Calcium, mg/dL	8.51 ± 0.43	8.57 ± 0.45	0.286
Magnesium, mg/dL	1.72 ± 0.22	1.73 ± 0.18	0.801
Albumin, g/dL	3.54 ± 0.91	3.87 ± 0.95	**0.010**
Total protein, g/dL	6.61 ± 0.79	6.77 ±0.78	0.128
CRP, mg/L	20.43 ± 9.59	19.86 ± 8.99	0.640
Total cholesterol, mg/dL	149.71 ± 46.63	164.33 ± 44.59	**0.015**
Triglyceride, mg/dL	164.21 ± 69.17	148.25 ± 71.96	0.093
HDL, mg/dL	41.33 ± 11.96	41.95 ± 13.24	0.722
LDL, mg/dL	101.30 ± 38.71	94.72 ± 38.23	0.195
Sodium, mEq/L	137.84 ± 5.69	138.26 ± 5.93	0.591
Potassium, mEq/L	4.40 ± 0.84	4.36 ± 0.87	0.698
AST, U/L	28.91 ± 15.80	31.23 ± 16.92	0.295
ALT, U/L	19.50 ± 9.62	21.42 ± 12.06	0.214
Uric aside, mg/dL	4.32 ± 1.05	4.23 ± 1.21	0.587
BNP, pg/mL	410–535	410–400	0.268
LVEF, %	53.73 ± 6.10	55.35 ± 3.83	**0.036**
LVEDD, mm	51.11 ± 4.26	49.31 ± 3.93	**0.001**
LVESD, mm	33.94 ± 3.41	33.51 ± 3.34	0.337
IVST, mm	11.76 ± 1.81	11.48 ± 1.74	0.243
PWT, mm	10.60 ± 1.55	10.51 ± 1.48	0.638
sPAP, mm Hg	32.50 ± 13.61	30.40 ± 10.98	0.171
Mitral e, cm/s	7.16 ± 1.01	7.22 ± 0.959	0.649

Abbreviations: ALT, alanine transaminase; AST, aspartate aminotransferase; BNP, brain natriuretic peptide; CRP, c-reactive protein; HDL, high-density lipoprotein; IVST, interventricular septum thickness; LDL, low-density lipoprotein; LVEDD, left ventricular end-diastolic diameter; LVESD, left ventricular end-systolic diameter; LVEF, left ventricular ejection fraction; PWD, posterior wall thickness; sPAP, systolic pulmonary artery pressure. Results in bold are significant (*p* < 0.05).

**Table 4:** Comparison of mental and malnutrition scores between groups.

	Presence of AF N = 71	Sinus Rhythm N = 287	*p*-Value
CONUT score	2.15 ± 1.68	1.39 ± 1.52	**<0.001**
Presence of GLIM malnutrition, *n* (%)	41 (57.7)	98 (34.1)	**<0.001**
GNRI	90.67 ± 14.71	96.58 ± 16.72	**0.007**
PNI	37.02 ± 5.51	38.09 ± 5.10	0.122
Barthel index	75.98 ± 24.90	87.02 ± 18.68	**<0.001**
FRAIL scale	1.61 ± 1.07	1.09 ± 0.97	**<0.001**
Pfeiffer Questionnaire	4.14 ± 2.35	3.18 ± 2.25	**0.002**
SARC-F score	3.56 ± 2.02	2.75 ±1.81	**0.001**
MMSE score	18.43 ± 6.09	20.70 ± 5.98	**0.005**

Abbreviations: CONUT, controlling nutritional status; GLIM, global leadership initiative on malnutrition; GNRI, geriatric nutritional risk index; MMSE, mini-mental state examination; PNI, prognostic nutritional index. Results in bold are significant (*p* < 0.05).

**Table 5:** Evaluation of variables according to GLIM and CONUT.

	GLIM (+) N = 139	GLIM (−) N = 219	*p*-Value	Low CONUT N = 72	High CONUT N = 286	*p*-Value
Presence of AF, *n* (%)	41 (29.5)	30 (13.7)	**<0.001**	29 (40.3)	42 (14.7)	**<0.001**
LVEF	55.12 ± 4.30	54.96 ± 4.49	0.737	55.11 ± 4.28	54.70 ± 4.93	0.489
Age, year	83.94 ± 6.55	81.93 ± 5.19	**0.002**	82.28 ± 5.38	84.41 ± 7.16	**0.020**
Male, *n* (%)	72 (51.8)	115 (52.5)	0.895	147 (51.4)	40 (55.6)	0.528
Barthel index	68.30 ± 22.64	95.31 ± 8.94	**<0.001**	93.12 ± 9.15	51.87 ± 20.02	**<0.001**
FRAIL scale	2.08 ± 0.81	0.63 ± 0.67	**<0.001**	0.87 ± 0.78	2.50 ± 0.73	**<0.001**
Pfeiffer Questionnaire	5.32 ± 1.94	2.13 ± 1.52	**<0.001**	2.61 ± 1.73	6.38 ± 1.75	**<0.001**
SARC-F score	4.10 ± 1.73	2.16 ± 1.57	**<0.001**	2.40 ± 1.57	4.93 ± 1.65	**<0.001**
MMSE score	15.11 ± 5.10	23.51 ± 4.04	**<0.001**	21.75 ± 5.35	14.26 ± 4.94	**<0.001**

Abbreviations: AF, atrial fibrillation; CONUT, controlling nutritional status; GLIM, global leadership initiative on malnutrition; LVEF, left ventricular ejection fraction; MMSE, mini-mental state examination. Results in bold are significant (*p* < 0.05).

**Table 6:** Comparison of dead and survivor groups with variables.

	Dead N = 22	Survivors N = 336	*p*-Value
GLIM, *n* (%)	13 (59.1)	126 (37.5)	**0.044**
CONUT score	2.00–3.25	1.00–2.00	**0.018**
GNRI	86.00–18.00	93.00–20.00	0.062
PNI	35.50–4.00	36.00–6.00	0.061
Barthel index	80.00–55.00	95.00–20.00	**0.002**
FRAIL scale	2.00–1.25	1.00–2.00	**0.032**
Pfeiffer Questionnaire	3.50–5.00	3.00–4.00	0.057
SARC-F score	3.00–3.00	2.00–2.00	0.233
MMSE score	16.50–11.25	23.00–10.00	**0.012**
Presence of AF, *n* (%)	12 (54.5)	59 (17.6)	**<0.001**
LVEF	51.50–7.25	55.00–6.00	**0.001**

Abbreviations: AF, atrial fibrillation; CONUT, controlling nutritional status; GLIM, global leadership initiative on malnutrition; GNRI, geriatric nutritional risk index; LVEF, left ventricular ejection fraction; MMSE, mini-mental state examination; PNI, prognostic nutritional index. Results in bold are significant (*p* < 0.05). Data are expressed as median (interquartile range).

**Table 7:** Factors affecting atrial fibrillation.

	B	OR	*p*	95%Cl	
				Lower	Upper
GFR	**0.015**	**1.015**	0.089	0.998	1.033
LVEF	−0.107	0.899	**0.002**	0.842	0.960
Presence of GLIM malnutrition	1.036	2.819	**<** **0.001**	1.613	4.927
Diabetes Mellitus	−0.843	0.430	0.324	0.080	2.301
Hypertension	−0.455	0.634	0.424	0.208	1.938
COPD	0.473	1.605	0.241	0.728	3.540
Albumin	−0.294	0.745	0.061	0.548	1.013

Abbreviations: GFR, glomerular filtration rate; GLIM, global leadership initiative on malnutrition; LVEF, left ventricular ejection fraction. Results in bold are significant (*p* < 0.05).

## Data Availability

All data generated or analyzed during this study are included in this article. Further inquiries can be directed to the corresponding author.
